# Cord Blood Derived CD4^+^CD25^high^ T Cells Become Functional Regulatory T Cells upon Antigen Encounter

**DOI:** 10.1371/journal.pone.0029355

**Published:** 2012-01-17

**Authors:** Elisabeth Mayer, Christina Bannert, Saskia Gruber, Sven Klunker, Andreas Spittler, Cezmi A. Akdis, Zsolt Szépfalusi, Thomas Eiwegger

**Affiliations:** 1 Department of Pediatrics and Adolescent Medicine, Medical University of Vienna, Vienna, Austria; 2 University of Zurich, Swiss Institute of Allergy and Asthma Research (SIAF), Davos, Switzerland; 3 Surgical Research Laboratories and Core Facility Flow Cytometry, Medical University of Vienna, Vienna, Austria; French National Centre for Scientific Research, France

## Abstract

Background: Upon antigen exposure, cord blood derived T cells respond to ubiquitous environmental antigens by high proliferation. To date it remains unclear whether these “excessive” responses relate to different regulatory properties of the putative T regulatory cell (Treg) compartment or even expansion of the Treg compartment itself.

Methods: Cord blood (>37 week of gestation) and peripheral blood (healthy controls) were obtained and different Treg cell subsets were isolated. The suppressive potential of Treg populations after antigen exposure was evaluated via functional inhibition assays ([^3^H]thymidine incorporation assay and CFSE staining) with or without allergen stimulation. The frequency and markers of CD4^+^CD25^high^FoxP3^+^ T cells were characterized by mRNA analysis and flow cytometry.

Results: Cord blood derived CD4^+^CD25^high^ cells did not show substantial suppressor capacity upon TCR activation, in contrast to CD4^+^CD25^high^ cells freshly purified from adult blood. This could not be explained by a lower frequency of FoxP3^+^CD4^+^CD25^high^cells or FOXP3 mRNA expression. However, after antigen-specific stimulation in vitro, these cells showed strong proliferation and expansion and gained potent suppressive properties. The efficiency of their suppressive capacity can be enhanced in the presence of endotoxins. If T-cells were sorted according to their CD127 expression, a tiny subset of Treg cells (CD4^+^CD25^+^CD127^low^) is highly suppressive even without prior antigen exposure.

Conclusion: Cord blood harbors a very small subset of CD4^+^CD25^high^ Treg cells that requires antigen-stimulation to show expansion and become functional suppressive Tregs.

## Introduction

Allergic disease in infancy has dramatically increased in the past decades [1], [2] and preventive strategies have become of rising interest. It is assumed that immune dysregulation is an early event which is measurable in the perinatal period, which is directly linked to the maturation of the immune system [3], [4], [5]. There are three time points to implement preventive strategies: before sensitization, after sensitization (but before disease) and at the onset of disease.

It has been considered a long time that the neonate is immunologically naïve and the development of specific immune responses is restricted to the period after birth. However, *in utero* exposure to environmental antigens has been documented in cord blood [6], [7], [8], [9] and amniotic fluid [10] which suggest that an intrauterine sensitization to allergens is possible. An increased proliferation and cytokine production of cord blood mononuclear cells in response to nutritive allergens, when the mother has been exposed, such as bovine beta-lactoglobulin (BLG) and aero-allergens has been described [4], [11], [12], [13]. The mechanisms that regulate the development of allergen-specific immune responses in the fetus and their relevance in protection or disease development have not been completely understood. In general, a linkage to intrauterine exposure with resulting T cell priming is assumed [12], [14], [15], [16].

Cord blood T cells are considered antigen-inexperienced cells of naïve phenotype (90% of CD3^+^ T cells express CD45RA^+^). Despite this naïve phenotype, responses to allergens do occur in cord blood and seem to be more pronounced than in peripheral blood [17]. Moreover, immune response of cord blood mononuclear cells differs markedly from peripheral blood mononuclear cells later in life and is accompanied by apoptosis. Reasons for this altered response to allergens in cord blood, could relate to a functionally immature state of the putative regulatory T cell (Treg) compartment or recent thymic emigrants [18]. Naturally occurring subpopulation of Treg cells maintain self-tolerance and prevent autoimmunity, inhibit rejection of transplants, prevent the induction of anti-tumor responses, play a role in allergen tolerance and regulate the immune response to infectious disease [19]. In adults, the peripheral Treg cell compartment consists of thymus-derived Treg cells [19] and inducible Treg cells [20]. Both subsets of Treg cells have been primarily defined as CD4^+^ T cells that express high levels of CD25 (IL-2Rα) and FoxP3, a Forkhead box P3 gene product. Functional suppressive studies in humans have revealed that the brightest expression of CD25 (CD4^+^CD25^high^ T cells) possess inhibitory potential [21]. Treg cells possess a T cell receptor (TCR) repertoire as broad as CD25^−^ T cells [22] and it is known that Tregs become suppressive after stimulation via the TCR. Once activated, they suppress in an antigen non-specific manner [23]. Similarly, FoxP3, a characteristic marker of regulatory activity might also be transiently expressed by effector T cells during activation. There is no highly specific Treg marker defined so far [24], [25]. Recent studies have demonstrated that down regulation of CD127, a specific receptor for IL-7 can distinguish Tregs from activated T cells [26], [27], [28].

In this study, we demonstrate that cord blood derived Treg possess the ability to become highly suppressive upon antigen exposure. Based on these results we hypothesize that early exposure to innocuous antigens is vital to develop T cell tolerance and is enhanced by innate immune response triggering environmental factors.

## Materials and Methods

### Ethics statement

The protocol was approved by the local ethical committee of the Medical University of Vienna and written informed consent was obtained from all participants of the study (protocol no EK 619/2006).

### Cell culture

Human umbilical cord blood from randomly chosen full term healthy infants (>37 weeks of gestation) was obtained by venopuncture of the umbilical vein immediately after delivery and placed in sterile sodium heparin tubes. Adult volunteers with no history of food allergy and no sensitization (specific IgE <0.35 kU/L, skin prick test negative) were used as controls. Heparinized blood was drawn over Ficoll-Paque (Pharmacia, Uppsala, Sweden). Mononuclear cells were isolated by density-gradient centrifugation as described [29]. All allergens used were tested for their endotoxin content present by using limulus amoeboid lysate assay (Cambrex Bio Science, Walkersville, MD, USA; limit of detection 0.035 EU/mL) [17], to get rid of possible confounders. The following BLG preparations were used as explained in detail in Eiwegger et al. (2008) [17]: BLG L-0130 (BLG [NP], Sigma Sciences, St Louis, MO, USA) were contaminated with endotoxins (118 EU/mL, >90% BLG) and LPS-free BLG (BLG [LF], PSDI-2400, produced from bovine whey protein isolate, Arla Food, Arhus, Denmark, >95% BLG] contained 0.96 EU. Purified, endotoxin-free Ara h 1, was provided by Dr E. N. Mills, (IFR Norwich, UK, <0.035 EU/mL). All assays were performed in serum-free Ultra Culture Medium (UCC, Biowhittaker, Walkersville, MD, USA) supplemented with 2 mM L-glutamine and 170 mg/L gentamycine-sulphate (Sigma Sciences) in 96-well round-bottom microplates in triplicates.

### In vitro proliferation assays

Proliferation was assessed via [^3^H]-thymidine incorporation assay and CFSE staining. The cells were cultured for 3 and 7 days and pulsed with [^3^H]-thymidine ([^3^H]TdR; 0.5 µCi [0.0185 MBq] per well; Amersham, U.K.) for the last 16 h of the culture. According to literature, a Stimulation index (SI) >2 was considered positive [30]. Cells were cultured either alone (control) or stimulated for 72 hours with 250 ng/mL plate-bound anti-CD3 (UCTH1 clone, Dako, Glostrup, Denmark) or 7 days with β-lactoglobulin (BLG [NP] or BLG [LF]).

#### Purification of regulatory T cells

The regulatory T cell subset (CD4^+^CD25^+^ cells) was generated via magnetic cell sorting (MACS®;Miltenyi Biotec, Auburn, CA, USA). CD4 T cells were negatively enriched using Miltenyi CD4 T cell isolation kit II (Miltenyi Biotec) according to the manufacturer's instruction. The CD25^+^ fraction was collected by eluting the cells two times through magnetic separation (MS) column to further enrich CD25^high^ cells. The CD4^+^CD25^−^ fraction was additionally purified over a LD column.

To investigate the relevance of CD127^low^ cells on the inhibitory capacity, CD4^+^ cells were negatively sorted via MACS® as described above and the respective subpopulations were isolated via a FACS sorter (Becton Dickinson, San José, CA, USA; FACSAria™; Purity >99.5%).

#### Inhibition assay

To assess the suppressive capacity of unprimed CD4^+^CD25^+^ T cells, increasing numbers of CD4^+^CD25^+^ T cells (2.5×10^3^–2.5×10^4^ cells/well) were co-cultured with CD4^+^CD25^−^ T cells (2.5×10^4^ cells/well). Cells were stimulated with 250 ng/mL plate-bound anti-CD3 (UCTH1 clone, Dako) or BLG [NP] [50 µg/mL] in the presence of irradiated autologous CBMCs or PBMCs (5×10^4^ cells/well; 3,000 rad). The cells were cultured for 3 or 6 days and pulsed with [^3^H]-thymidine for the last 16 h of the culture. The inhibitory potential is expressed as relative proliferation compared to the CD4^+^CD25^−^ cells.

#### Priming assay

CD4^+^CD25^−^ T cells were MACS-sorted and frozen on day 0. CBMCs were first stimulated for 6 days in the presence of LPS-free-BLG [LF] [50 µg/mL] or BLG [NP] ([50 µg/mL]; in a 25 cm^2^ culture flask (BD Falcon).

### Flow Cytometric analysis

Four color fluorescence staining was performed using anti-CD3 (PerCP, APC), anti-CD8 (PE, PerCP), anti-CD25 (APC), anti-CD45RA (PE), anti-CD45RO (PE, APC), anti-CD45RB (PE), anti-CD69 (PE), anti-CD103 (PE), anti-CD127 (PE), anti-HLA-DR (PE) and anti-CTLA-4 (PE), as well as regarding isotype controls (all from Becton Dickinson) and anti-CD25 (PE) (Miltenyi Biotec, Bergisch Gladbach), CD127 (APC) (R&D, McKinley Place, MN, USA), GARP (Alexis Corporation, Farmingdale, NY, USA) and a PE anti-mouse/rat FoxP3 Staining Set (e-Biosciences, San Diego, CA) and were used according to the manufacturer's instructions.

To define dividing cells, purified cell fractions were stained with carboxyfluorescein diacetate succinimidyl ester (CFSE, Sigma-Aldrich) as explained recently [17].

Cord blood derived CD4^+^CD25^+^ and CD4^+^CD25^−^ cells were isolated with the Treg isolation kit (Miltenyi Biotec) stained with CFSE according to the manufacturer's protocol. CD4^+^CD25^+^ cells alone and CD4^+^CD25^−^ cells alone as well as a combination of CD4^+^CD25^+^ and CD4^+^CD25^−^ where always only one population was CFSE stained, was incubated with different stimuli (BLG [NP], BLG [LF]) and medium control for 7 days in a CO_2_ incubator. On day 7, cells were analyzed via a cytometer and proliferation was measured.

All cytometric measurements were performed with a FACS Calibur and evaluated with CellQuest software (Becton Dickinson).

### PCR

Total RNA was extracted from freshly isolated CBMCs or PBMCs using the GenElute Mammalian Total RNA Miniprep Kit (Sigma Sciences). The RNA was reverse-transcribed applying “iScript cDNA Synthesis Kit” from Bio-Rad (Hercules, USA) according to the manufacturer's instructions. Real-time polymerase chain reaction (RT-PCR) amplification was performed in triplicate on CFX96 (Bio-Rad) using a cycling profile of 2 min at 95°C followed by a total of 40 temperature cycles with 15 sec at 95°C and 1 min at 60°C. All experiments were run in triplicates with the same thermal-cycling parameters. Expression of FOXP3, IL-10, TGF-β, GARP (FOXP3 FW 5′-ATG GCC CAG CGG ATG AG-3′, RV 5′- GAA ACA GCA CAT TCC CAG AGT TC -3′, IL10 FW 5′- GTG ATG CCC CAA GCT GAG A -3′, RV 5′- CAC GGC CTT GCT CTT GTT TT -3′, GARP FW 5′-GCC CTG TAA GAT GGT GGA CAA G -3′, RV 5′-CAG ATA GAT CAA GGG TCT CAG TGT CT -3′ and r18S (housekeeping gene) were measured using QuantiTect primers (Qiagen, Hilden, Germany). Melting-curve analysis was used to assess the specificity of the assay. To compare expression of genes between patient groups the calculation was as follows: The sample with the lowest amount of the respective gene was set as internal standard to one and relative expression was calculated as follows: 2^−(ΔCt sample of interest−ΔCt lowest sample)^.

### Statistical analysis

Statistical analysis was performed with SPSS software (version 16 for Windows; SPSS, Inc, Chicago, III). Groups were compared using the Mann-Whitney U test for (non-parametric) unpaired responses. The Wilcoxon matched-pairs signed-rank test was used to examine paired data. When Gaussian distribution was given, the Student t-test for independent samples or matched pairs t-test was performed. A p-value of less than 0.05 was considered statistically significant for all analyses.

## Results

### Comparable Treg-related transcription factor and marker expression in cord and peripheral blood

**Figure 1:**
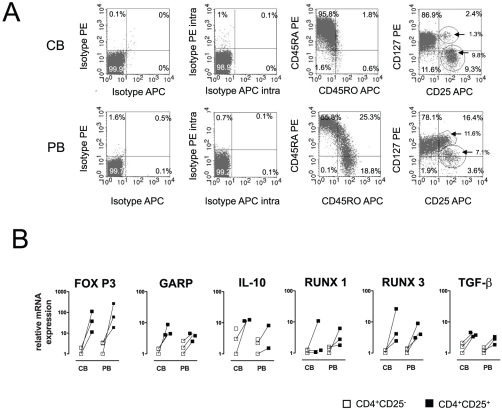
Comparable Treg-related transcription factor and marker expression in CB and PB on day 0. (**A**) Cord and peripheral blood derived mononuclear cells were analyzed via flow cytometry on day 0 without prior stimulation. Surface and intracellular staining was performed and one out of three representative experiments is shown. (**B**) Real-time PCR analysis was performed to quantify the gene expression of Treg-related markers (FOXP3, GARP, TGF-β, RUNX 1, RUNX 3, and IL-10). Results are representative for three independent experiments showing the normalized expression. CD4^+^CD25^−^ T cells are displayed with white and CD4^+^CD25^+^ T cells with black squares.

Cord blood varies from PB due to a differential surface expression. In CB, about 90%±8.5 (Mean±SD) T helper cells are CD45RA^+^ whereas only 54%±3.5 of the PB derived T cells show a naïve phenotype. In addition, the CD127 pattern is also different in the PB compartment. A higher amount of CD127^+^ T cells (6.5%±1.6 vs 2.7±2.4) but a similar amount of CD127^low^CD25^+^ T cells was observed (6.3±1.4 vs 8.2±1.8); **Figure 1A**).

Moreover, FoxP3-expression, the most important transcription factor in Tregs has been examined by intracellular staining and flow cytometry. Gating strategy was performed according to several published approaches but no significant differences could be detected (supporting information, **Figure S1**).

Messenger RNA expression of a panel of Treg-associated transcription factors and Treg-associated cytokines was measured in the CD25 positive and negative fraction in cord blood and peripheral blood. GARP, RUNX1, RUNX3, TGF-β, IL-10 mRNAs were determined. There was a strong up-regulation of these markers in the CD25 positive fraction, however there were no significant differences with regard to the relative up-regulation of these Treg-specific markers between cord blood and peripheral blood (**Figure 1B**).

### Increased T cell proliferative responses to BLG in cord blood is due to a significantly reduced suppressive capacity of CD4^+^CD25^+^ cells

**Figure 2:**
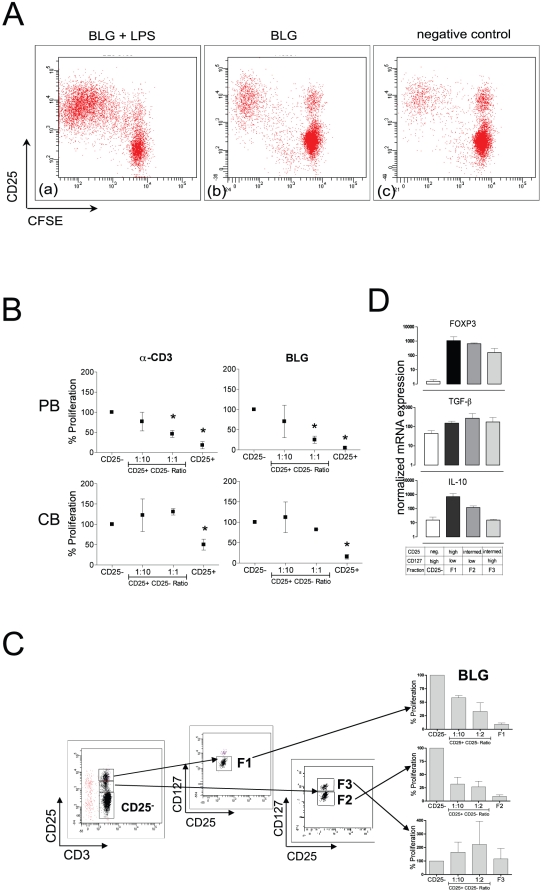
CB-derived CD4^+^CD25^+^ T cells are deficient with regard to their regulatory function harboring a subset with limited net suppressive effects. (**A**) Specificity of proliferation to BLG and BLG+LPS is displayed via CFSE labeling experiments. (**B**) Inhibition experiments were performed using and increasing amount of MACS-sorted CD4^+^CD25^+^ T cells to the CD4^+^CD25^−^ fraction in presence of irradiated APCs. The inhibitory potential is expressed as relative proliferation compared to the CD4^+^CD25^−^ cells. Graphs indicate the means of 5–8 independent experiments with means +/− SEM. Wilcoxon sign rank test was applied. P-values of less than 0.05 were considered significant. (**C**) CBMCs were separated via FACS on day 0 according to their CD25 and CD127 expression. Four subgroups (CD4^+^CD25^high^CD127^low^ (F1), CD4^+^CD25^intermediate^CD127^low^ (F2), CD4^+^CD25^intermediate^CD127^high^ (F3), and CD4^+^CD25^−^ T cells (CD25^−^)) were obtained. An inhibition assay was performed with the first three fractions using the CD4^+^CD25^−^ T cells as effector cells. The inhibitory potential is expressed as relative proliferation compared to the CD4^+^CD25^−^ subgroup. Graphs indicate the means of 4 independent experiments and SEM. (**D**) Real-time PCR analysis was performed to analyze the FOXP3, TGF-β, and IL-10 expression for further examination of the four fractions. Data represents three independent experiments showing the normalized expression of means +/− SEM.

Higher T cell proliferation has been observed in cord blood-derived mononuclear cells compared to peripheral blood-derived cells in response to one of the major milk allergens BLG [17]. To assess whether the reasons for this altered responses in cord blood could be explained by differences in regulatory T cells in cord blood, the inhibitory capacity of CB derived Tregs were compared to those from adult blood. The unprimed PB-derived CD4^+^CD25^+^ fraction from adults (**Figure 2A**) and children under the age of four (supporting information, **Figure S2**) possessed dose-dependent inhibitory properties, whereas CB-derived CD4^+^CD25^+^ T-cells were hypoproliferative but significantly less potent.

### The CD25^+^CD127^low^ compartment of CD4^+^ T cells harbors a regulatory T cell subset with limited net suppressive effect

No or low CD127 expression has been described to be an excellent marker for Treg characterization [31]. Recently, the suppressive capacity of CD4^+^CD25^+^CD127^low/−^ Tregs in cord blood has been linked to the development of food allergy [32]. The CD127^low^ fraction was purified by means of FACS-sorting from the CD25^high^ and CD25^med^ fraction to investigate the suppressive capacity of low CD127 expressing Treg cells in cord blood.

Subsets with low CD127 expression (CD4^+^CD25^high^CD127^low^ (**Figure 2B**; F1) and CD4^+^CD25^int^CD127^low^ (**Figure 2B**; F2) possessed suppressive potential, whereas the CD4^+^CD25^int^CD127^high^ subset (**Figure 2B**; F3) was associated with proliferation upon BLG stimulation. The same effect has been observed upon stimulation with the allergens Ara h 1 and OVA (supporting information, **Figure S3**). TGF-β or IL-10 mRNA levels were not substantially up-regulated whereas FOXP3 was increased in the respective subsets (**Figure 2C**).

### Cord blood-derived CD4^+^CD25^+^ T cells become highly suppressive

**Figure 3:**
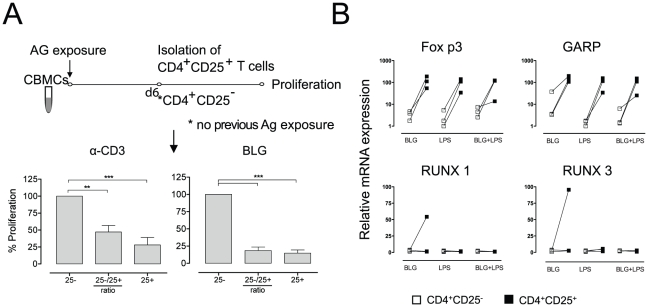
Upon priming with BLG, cord blood derived CD4^+^CD25^+^ T cells become highly suppressive. (**A**) Cord blood-derived mononuclear cells were co-cultured for 6 days with the antigen BLG. At day six CD4^+^CD25^+^ T cells were MACS-sorted and an inhibition assay was performed with the CD4^+^CD25^−^ (frozen on day 0, without prior antigen exposure) and stimulated with α-CD3 or BLG. The inhibitory potential is expressed as relative proliferation compared to the CD4^+^CD25^−^ cells. Results are representative for 6–9 independent experiments. Wilcoxon signed rank test was applied. P-values of less than 0.05 were considered significant (*); p<0.005 (**); p<0.001 (***). (**B**) Real-time PCR analysis was performed to quantify the gene expression of Treg-related markers (FOXP3, GARP, RUNX 1, and RUNX 3) after allergen exposure. Results are representative for three independent experiments showing the normalized expression. CD4^+^CD25^−^ T cells are displayed with white and CD4^+^CD25^+^ T cells with black squares.

Based on the fact, that tolerance development after birth has to take place within a short period of time the role of environmental antigens to prime this procedure was investigated [33]. CBMCs were exposed for six days to BLG in the presence or absence of LPS (**Figure 3A**). CD4^+^CD25^+^ cells were isolated and their suppressive potential on anti-CD3- and BLG-induced proliferation of autologous CD4^+^CD25^−^ cells was investigated, and an inhibition of 53% and 83%, respectively, was observed. The same regulatory subset that did not show any inhibitory function in the experiments on day 0 gained inhibitory potential after stimulation with BLG. FOXP3 and GARP expression was strongly up regulated in Tregs when compared to CD4^+^CD25^−^ T cells. BLG exposed cells displayed relatively higher amounts FOXP3 and GARP. The expression pattern of RUNX 1 and RUNX 3, however, is less clear. A trend towards a higher expression of RUNX 1 and RUNX 3 is observed in cord blood mononuclear cells (**Figure 3B**).

### Evidence for activation and expansion of Tregs in cord blood upon exposure to allergens

**Figure 4:**
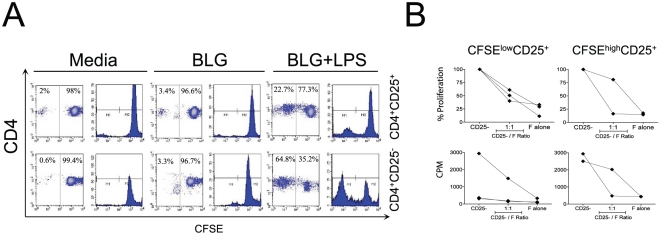
Evidence for activation and expansion of Tregs in cord blood upon exposure to allergens. (**A**) CD4^+^CD25^+^ and CD4^+^CD25^−^ T cells were isolated via MACS on day 0, stained with CFSE and stimulated for 7 days in the presence of medium alone (left), BLG (middle), and BLG+LPS (right). Cells were gated on CD3^+^CD4^+^ cells and proliferation was measured by rating the CFSE distribution. For every stimulus a scatter dot-plot (left) and a histogram (right) is displayed. Data represents one out of four independent experiments. Cord blood derived CD4^+^CD25^+^ T cells do proliferate upon BLG+LPS stimulation (22.65%), and to a lesser extent when stimulated with BLG (3.37%). In contrast, CD4^+^CD25^−^ T cells strongly proliferate upon stimulation with BLG+LPS (64.79%). (**B**) After a six-day priming with BLG, cells were FACS sorted according to their CFSE intensity and CD25 expression. Inhibition assays were performed with CFSE^low^CD25^+^ and CFSE^high^CD25^+^ T cells representing the regulatory fraction and with antigen-inexperienced CD4^+^CD25^−^ in the presence of BLG. The relative inhibition and the counts per minute are displayed.

*In vitro*, allergen stimulated CBMC displayed potent suppressive functions. Thus, it was of interest to elucidate whether this is due to the switch and generation of new or expansion of pre-existing antigen-specific regulatory T cells. Initially, CD4^+^CD25^+^ and CD4^+^CD25^−^ from CB were MACS-sorted, CFSE-labeled on day 0 and then were stimulated with BLG (middle), or BLG+LPS (right) (**Figure 4A**). As anticipated the CD4^+^CD25^+^ fraction was hypoproliferative as compared to the CD4^+^CD25^−^ fraction. However, in cord blood the CD25^−^ fraction down regulated CD4 as descried in apoptotic, cord blood derived CD4^+^ T-cells.

Thereafter, cells were cultured with the allergen and sorted according to their CFSE intensity and CD25 expression on day 6 as indicated in **Figure S4** (supporting information). Again, inhibition assays were performed (**Figure 4B**). Importantly both, the dividing and the non-dividing fractions emerging from the CD4^+^CD25^+^ cell population, possessed equal suppressive capacity after the allergen exposure

## Discussion

The allergen specific immune response in CB is poorly understood and specificity of allergen proliferation regarding the detection of clinically relevant effector T cells is still questioned. We could recently demonstrate that CB derived mononuclear cells are more sensitive than mononuclear cells from adults in terms of proliferative responses to both, purified allergens and allergens plus endotoxin [17], [34]. As human CB mainly harbors naïve T cells they represent a perfect model system for a naïve human system to assess the impact of allergens.

Allergen-specific proliferative responses in CB vs. adult blood cells were significantly higher quantitatively and qualitatively (supporting information, **Figure S5**). We provide evidence that freshly isolated CB derived Tregs share many phenotypic characteristics with their PB derived counterpart. They lack however potent suppression of CD4^+^CD25^−^ T cells, in contrast to PB derived Tregs. This is observed both for TCR-cross-linking and allergen-specific stimulation. Although our finding are consistent with findings of many different groups [18], [35], [36], [37], [38], [39] the herein provided data is focusing on the antigen specific subsets. Although some studies demonstrated a strong suppressive potential of CB derived Tregs, at a closer look this was obtained after pre-activation with polyclonal stimuli [40], [41]. This was also observed in our hands upon expansion of CD4^+^CD25^+^ T cells via CD3CD28 beads in the presence of IL-2. The reasons for this reduced functionality remains to be speculative. Several points can be ruled out by our experiments. First, no significantly lower FoxP3^+^CD4^+^CD25^high^ T cells numbers are observed in cord blood. Second, the regulatory T cell fraction CD4^+^CD25^+^CD127^low^ possessed inhibitory capacity without pre-activation as indicated but does not explain the cord blood related differences. Third, CB and PB derived mononuclear cells mainly differ in their percentage of naïve T cells that express a CD45RA^+^CD45RO^−^CD62L^bright^ phenotype whereas the adult CD4^+^ T cells exhibit a memory phenotype (CD45RA^−^CD45RO^+^) [21], [42]. So it is conceivable that freshly isolated CB CD4^+^CD25^+^ T cells are less suppressive due to their phenotypic immaturity.

Based on numerous studies performed with Tregs, it is possible that a specific subset of naïve CB CD4^+^CD25^−^ can be “educated” and expanded to become suppressive CD25^+^FoxP3^+^ Tregs as also suggested by Zelenay et al [43]. Our study demonstrates the inhibitory potential of the regulatory T cell subset after stimulation *in vitro* with allergens. The newly generated Tregs were highly suppressive in an antigen-nonspecific manner. This corresponds with the observation in other studies as CD25^+^ Tregs need antigen specific stimulation via the TCR to become suppressive, but once activated, they suppress antigen-unspecifically [23]. Thornton et al. (2004) also posted that the CD4^high^CD25^+^ population is a surviving population in cord blood. We focused in particular on these populations.

Based on our findings, we hypothesize that endotoxins and also allergen contact directly after birth are pivotal to gain allergen specific suppressive regulatory functions upon re-exposure resulting in tolerance to environmental antigens. This is in line with shift in the paradigm from allergen avoidance direction exposure in order to achieve tolerance. In fact no association between the level of environmental exposure and the extent of allergen-induced proliferation could be found [44]. The exposure is also not related to an increased sensitization [45]. The mechanism to acquire tolerance is not clear yet but regarding our mRNA data we can say that it cannot be explained due to TGF-β or IL-10 related effects. The extent of TGF-β or IL-10 up-regulation however is by far less than for FOXP3.

In summary, we have shown that cord blood derived Tregs need to be pre-activated before becoming highly suppressive. Regulatory subsets are generated by both, functional maturation and clonal expansion.

## Supporting Information

Figure S1 **FoxP3 expression was analyzed comparing two gating strategies.** For the Gating strategy 1 the percentages of CD3^+^CD4^+^CD25^+^FoxP3^+^ cells are included whereas for the Gating strategy 2 the top 2% of the CD4^+^CD25^+^ T cells are gated to visualize the FoxP3 expression (CB = white bars; PB = black bars; mean+/−SEM). CFSE stained CBMCs were incubated in a culture flask (25 cm^2^) for 6 days in the presence of BLG+LPS, BLG, and with medium alone (negative control). The CFSE and CD25 expression was analyzed via flow cytometry.
(DOCX)

Figure S2 **PBMCs of children with a mean age of four years were obtained on day 0 and the putative Treg fraction (CD4^+^CD25^+^ T cells) was isolated via MACS.** Inhibition experiments were performed and the inhibitory potential is expressed as relative proliferation compared to CD4^+^CD25^−^ cells. Graphs indicate the means of 18 independent experiments and SEM. Wilcoxon sign rank test was applied. P-values of less than 0.05 were considered significant.
(DOCX)

Figure S3 **CBMCs were FACS sorted on day 0 according to their CD25 and CD127 expression.** Four subgroups (CD4^+^CD25^high^CD127^low^ (F1), CD4^+^CD25^intermediate^CD127^low^ (F2), CD4^+^CD25^intermediate^CD127^high^ (F3), and CD4^+^CD25^−^ T cells (CD25^−^)) were obtained. An inhibition assay was performed with the three fractions using the CD4^+^CD25^−^ T cells as effector cells in the presence of the peanut allergen Ara h 1 and the egg allergen Ovalbumin (OVA). The inhibitory potential is expressed as relative proliferation compared to the CD4^+^CD25^−^. Graphs indicate the means of 2–4 independent experiments and SEM.
(DOCX)

Figure S4 **After a six-day stimulation with BLG, cells were FACS-sorted according to their CFSE intensity and CD25 expression.**
(DOCX)

Figure S5 **Proliferative response of CBMCs (Cord blood mononuclear cells) to BLG is significantly increased on day seven compared to peripheral blood derived mononuclear cells (PBMCs) of adult individuals.** (cord blood: n = 17; peripheral blood: n = 10). Graph indicates the mean and the standard error of the mean. Mann Whitney U-Test was applied. P-values of less than 0.05 were considered significant.
(DOCX)
