# Supplementary material for: Cord Blood Derived CD4+CD25high T Cells Become Functional Regulatory T Cells upon Antigen Encounter
Source: PLoS One. 2012 Jan 17;7(1):e29355. doi: 10.1371/journal.pone.0029355 (PMC3260151; doi:10.1371/journal.pone.0029355)
Supplement: Figure S1 — FoxP3 expression was analyzed comparing two gating strategies. For the Gating strategy 1 the percentages of CD3+CD4+CD25+FoxP3+ cells are included whereas for the Gating strategy 2 the top 2% of the CD4+CD25+ T cells are gated to visualize the FoxP3 expression (CB = white bars; PB = black bars; mean+/−SEM). CFSE stained CBMCs were incubated in a culture flask (25 cm2) for 6 days in the presence of BLG+LPS, BLG, and with medium alone (negative control). The CFSE and CD25 expression was analyzed via flow cytometry. (DOCX) [file pone.0029355.s001.docx]

-
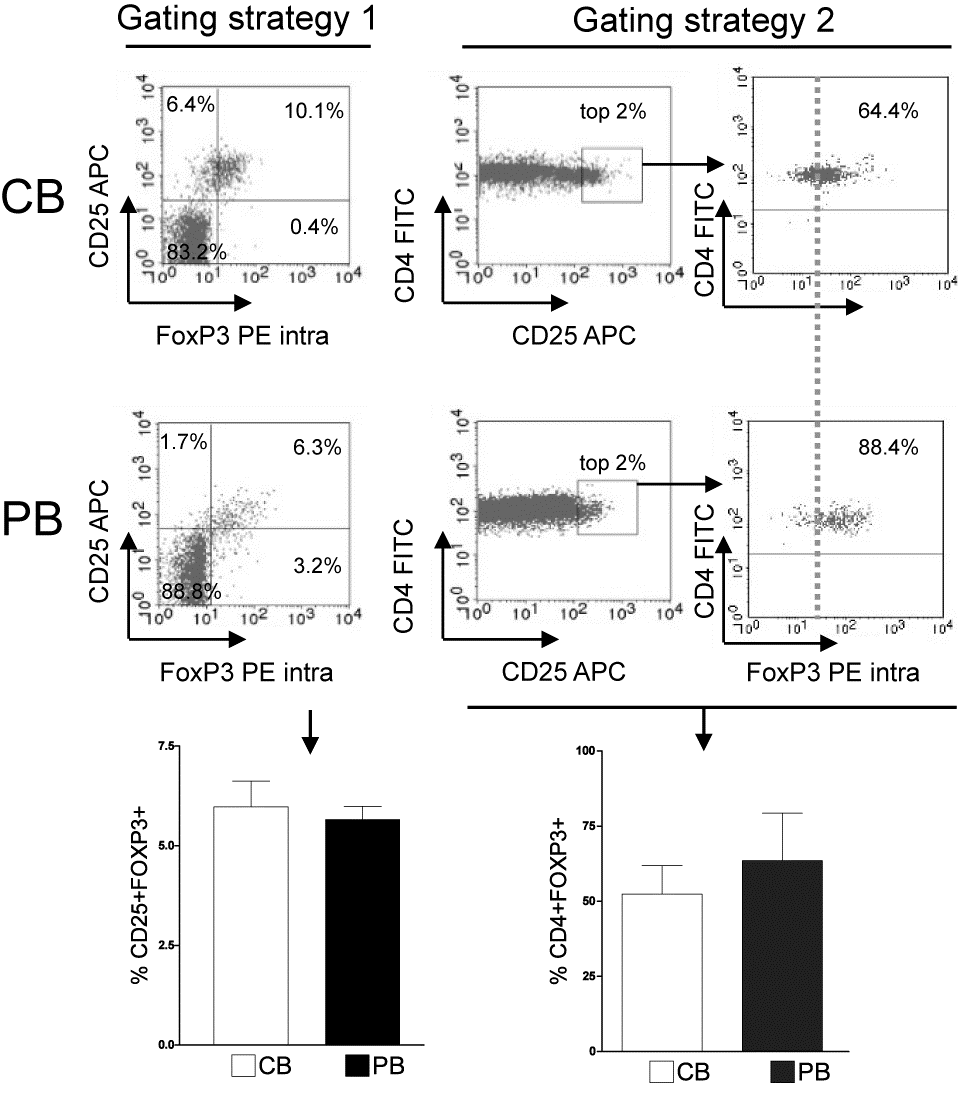


Figure S1

FoxP3 expression was analyzed comparing two gating strategies. For the Gating strategy 1 the percentages of CD3^+^CD4^+^CD25^+^FoxP3^+^ cells are included whereas for the Gating strategy 2 the top 2% of the CD4^+^CD25^+^ T cells are gated to visualize the FoxP3 expression (CB = white bars; PB = black bars; mean+/-SEM).

CFSE stained CBMCs were incubated in a culture flask (25 cm^2^) for 6 days in the presence of BLG+LPS, BLG, and with medium alone (negative control). The CFSE and CD25 expression was analyzed via flow cytometry.
