# Supplementary material for: Cord Blood Derived CD4+CD25high T Cells Become Functional Regulatory T Cells upon Antigen Encounter
Source: PLoS One. 2012 Jan 17;7(1):e29355. doi: 10.1371/journal.pone.0029355 (PMC3260151; doi:10.1371/journal.pone.0029355)
Supplement: Figure S2 — PBMCs of children with a mean age of four years were obtained on day 0 and the putative Treg fraction (CD4+CD25+ T cells) was isolated via MACS. Inhibition experiments were performed and the inhibitory potential is expressed as relative proliferation compared to CD4+CD25− cells. Graphs indicate the means of 18 independent experiments and SEM. Wilcoxon sign rank test was applied. P-values of less than 0.05 were considered significant. (DOCX) [file pone.0029355.s002.docx]

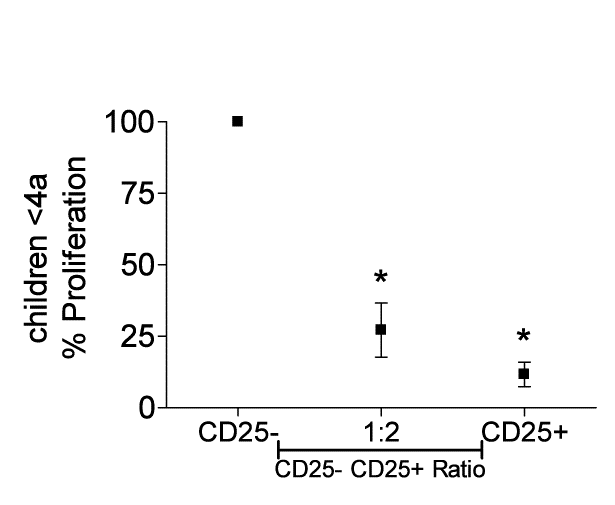


Figure S2

PBMCs of children with a mean age of four years were obtained on day 0 and the putative Treg fraction (CD4^+^CD25^+^ T cells) was isolated via MACS. Inhibition experiments were performed and the inhibitory potential is expressed as relative proliferation compared to CD4^+^CD25^-^ cells. Graphs indicate the means of 18 independent experiments and SEM. Wilcoxon sign rank test was applied. P-values of less than 0.05 were considered significant.
