# Supplementary material for: Cord Blood Derived CD4+CD25high T Cells Become Functional Regulatory T Cells upon Antigen Encounter
Source: PLoS One. 2012 Jan 17;7(1):e29355. doi: 10.1371/journal.pone.0029355 (PMC3260151; doi:10.1371/journal.pone.0029355)
Supplement: Figure S3 — CBMCs were FACS sorted on day 0 according to their CD25 and CD127 expression. Four subgroups (CD4+CD25highCD127low (F1), CD4+CD25intermediateCD127low (F2), CD4+CD25intermediateCD127high (F3), and CD4+CD25− T cells (CD25−)) were obtained. An inhibition assay was performed with the three fractions using the CD4+CD25− T cells as effector cells in the presence of the peanut allergen Ara h 1 and the egg allergen Ovalbumin (OVA). The inhibitory potential is expressed as relative proliferation compared to the CD4+CD25−. Graphs indicate the means of 2–4 independent experiments and SEM. (DOCX) [file pone.0029355.s003.docx]

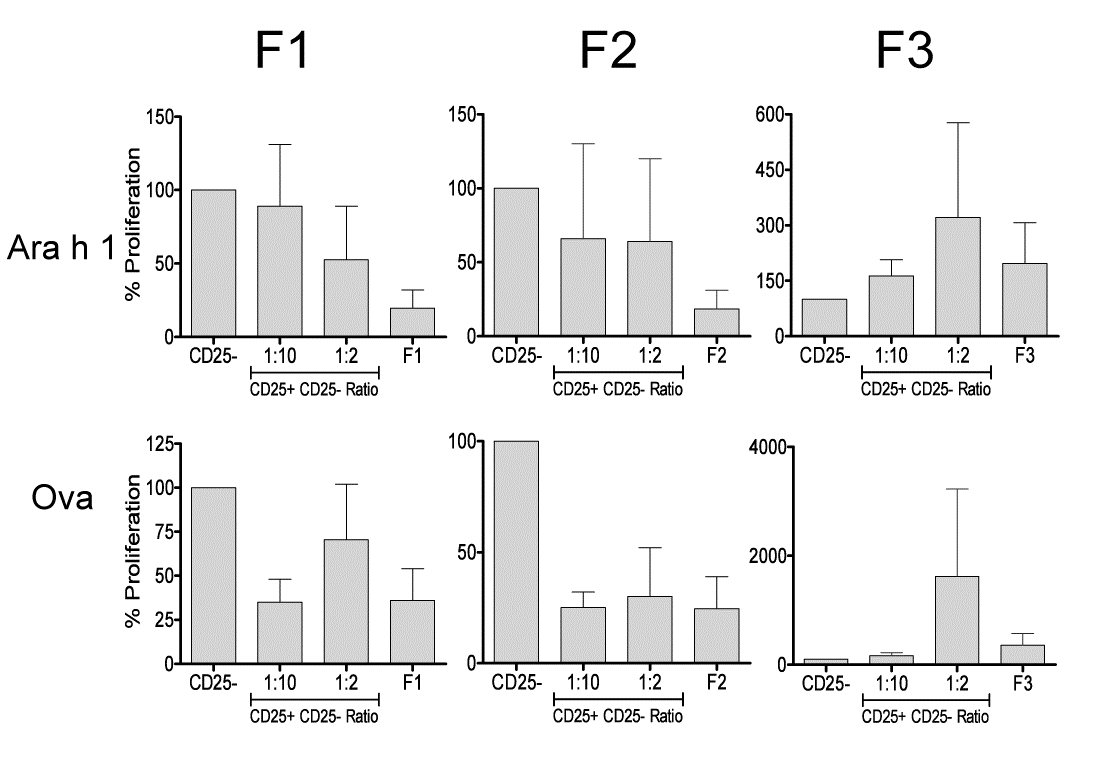


Figure S3

CBMCs were FACS sorted on day 0 according to their CD25 and CD127 expression. Four subgroups (CD4^+^CD25^high^CD127^low^ (F1), CD4^+^CD25^intermediate^CD127^low^ (F2), CD4^+^CD25^intermediate^CD127^high^ (F3), and CD4^+^CD25^-^ T cells (CD25^-^)) were obtained. An inhibition assay was performed with the three fractions using the CD4^+^CD25^-^ T cells as effector cells in the presence of the peanut allergen Ara h 1 and the egg allergen Ovalbumine (OVA). The inhibitory potential is expressed as relative proliferation compared to the CD4^+^CD25^-^. Graphs indicate the means of 2-4 independent experiments and SEM.
