# Supplementary material for: Cord Blood Derived CD4+CD25high T Cells Become Functional Regulatory T Cells upon Antigen Encounter
Source: PLoS One. 2012 Jan 17;7(1):e29355. doi: 10.1371/journal.pone.0029355 (PMC3260151; doi:10.1371/journal.pone.0029355)
Supplement: Figure S4 — After a six-day stimulation with BLG, cells were FACS-sorted according to their CFSE intensity and CD25 expression. (DOCX) [file pone.0029355.s004.docx]

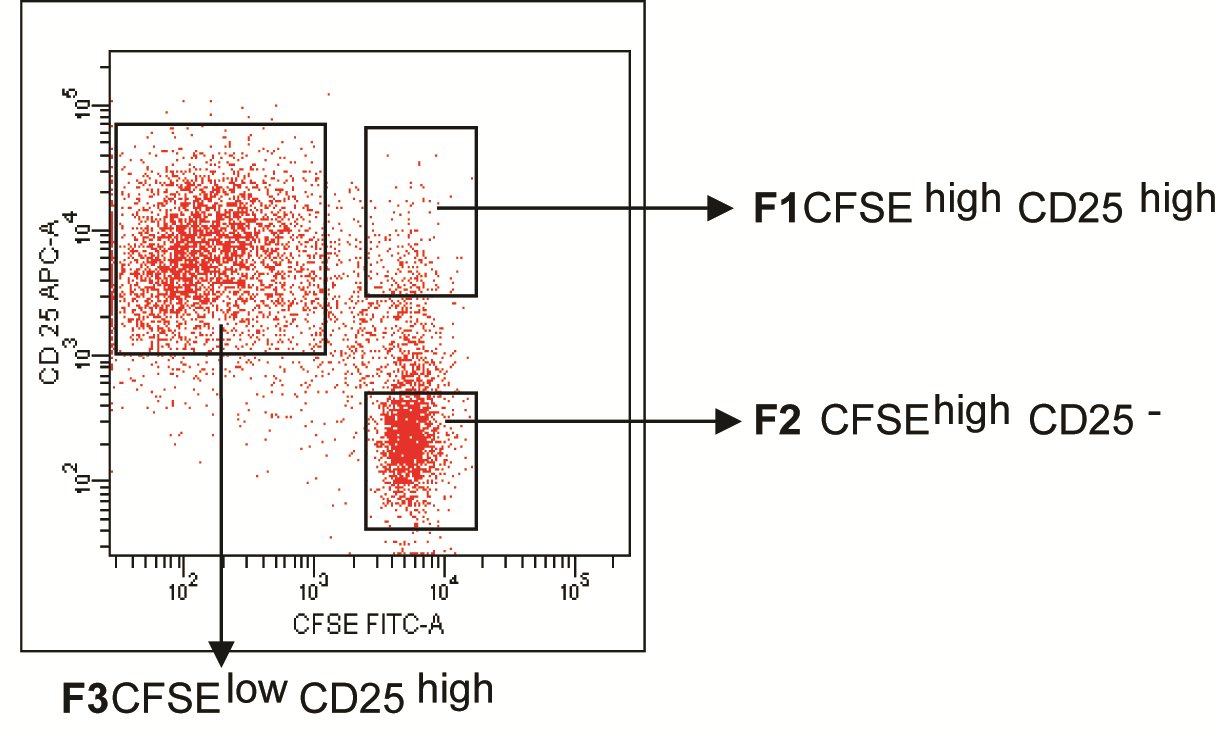


**Figure S4**

After a six-day stimulation with BLG, cells were FACS-sorted according to their CFSE intensity and CD25 expression.
