# Supplementary material for: Cord Blood Derived CD4+CD25high T Cells Become Functional Regulatory T Cells upon Antigen Encounter
Source: PLoS One. 2012 Jan 17;7(1):e29355. doi: 10.1371/journal.pone.0029355 (PMC3260151; doi:10.1371/journal.pone.0029355)
Supplement: Figure S5 — Proliferative response of CBMCs (Cord blood mononuclear cells) to BLG is significantly increased on day seven compared to peripheral blood derived mononuclear cells (PBMCs) of adult individuals. (cord blood: n = 17; peripheral blood: n = 10). Graph indicates the mean and the standard error of the mean. Mann Whitney U-Test was applied. P-values of less than 0.05 were considered significant. (DOCX) [file pone.0029355.s005.docx]

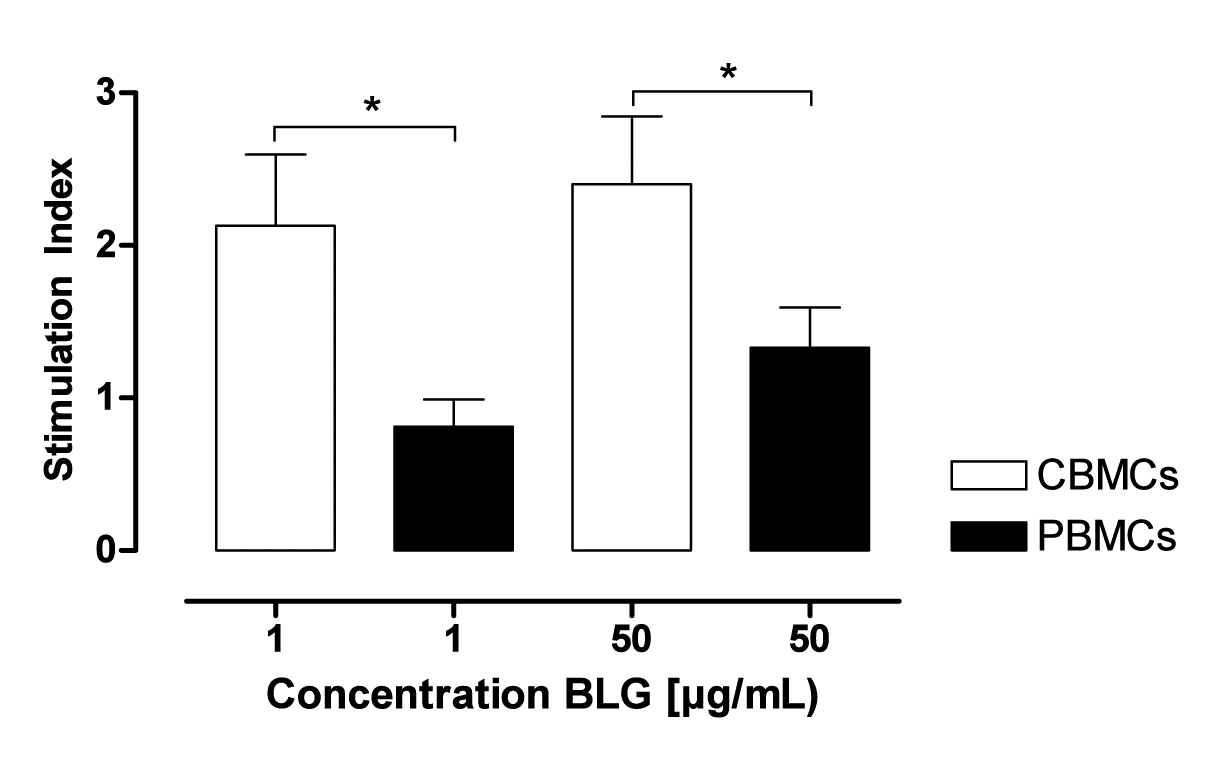


**Figure S5**

Proliferative response of CBMCs (Cord blood mononuclear cells) to BLG is significantly increased on day seven compared to peripheral blood derived mononuclear cells (PBMCs) of adult individuals.

(cord blood: n=17; peripheral blood: n=10). Graph indicates the mean and the standard error of the mean. Mann Whitney U-Test was applied. P-values of less than 0.05 were considered significant.
